# Midwifery

**Published:** 1826-04

**Authors:** 


### MIDWIFERY.

40. *A contribution to the History of the development of the human Embryo in the first three weeks after the conception.* By Dr. POCKELS, of Brunswick.

The following interesting contribution to the history of the early development of the human embryo, is extracted from the *Isis* of Dr. Oken for December last. The description here given is accompanied in the Isis with copper-plate engravings exhibiting various views of the newly discovered structure magnified, and of the natural size. Through the courtesy of Dr. Pockels we have been favoured with a sight of the preparations themselves; they correspond perfectly with the description given, and are put up with a neatness and ingenuity to be equalled only in the museums of the Dutch.

Out of more than fifty human ova aborted during the first six weeks of pregnancy which I had a few years since (says Dr. P.) an opportunity of examining, I obtained four perfectly normal, which were voided between the eighth and sixteenth day after conception. The examination was made under water in a flat black dish, to the bottom of which the ovum was made to adhere that it might not be disturbed by the movement of the water, and for the purpose of allowing of a more accurate inspection with the magnifying glass.

The results of this investigation are for the most part new and interesting in the history of the development of the human ovum. They are, in my opinion, sufficiently clear to render a more detailed demonstration unnecessary, and I shall therefore give in the following paragraphs a short account of the matter.

The naturally formed ovum within the first fourteen days after conception is about the size of a nutmeg or of a small walnut; it lies with the surface of the chorion imbedded in the tunica decidua of Hunter, and is easily taken out of this situation, after cutting through the decidua, without being torn. There is no vascular connexion between the two surfaces. The cavity of the chorion contains a somewhat reddish transparent fluid about the consistence of the white of an egg, and a delicate colourless web traverses the fluid in different directions, very similar to the structure of the vitreous humour of the eye. I could never discover any traces of an allantois on the inner surface of the chorion.

In this albuminous-like fluid of the chorion lies the amnion, in the first fourteen days after conception, of about the size of a pea or a field bean, generally pear-shaped, but sometimes round. Through defect of practice in the examination it is very easy to separate or throw away the amnion with the albuminous fluid of the chorion, and one supposes that he has found an ovum without an embryo; this, I must confess, formerly happened to myself. For to expose the ovum properly, the albumen and the transparent web-like texture of the chorion is to be taken away, and then the small white body of the embryo with its connected organs, and the amnion vesicle are brought into view. The amnion is usually fastened slightly by its pear-shaped peduncle to a fronticular part of the chorion, and projects with its circular portion into the albumen: its coats are transparent, and it contains a transparent fluid.

The embryo appears to the naked eye in even fourteen days old, as a whitish yellowish body, scarcely a line thick, flat in the middle and compressed, at both ends thick and irregularly rounded, and has the consistence of jelly. The embryo lies until about the twelfth day after conception, external to the cavity of the amnion with its back in a superficial depression on the outer surface of the amnion, and attached to it by a transparent cellular web, so slightly that it allows of being raised from the amnion without opening that vesicle. Its belly side is turned toward the chorion. About the eighth day the embryo increasing in size with its back still towards the amnion, presses the latter structure downwards, and thus gradually forms for itself a vagina on its belly side out of the amnion, just as in the fœtus the testicle forms a covering for itself of peritonæum as it descends through the internal abdominal ring. Towards the sixteenth day the embryo lies in the amnion close on the inner surface of this vesicle, but yet without an umbilical cord, during the evolution the embryo goes farther into the amnion, and the vagina on the belly-side of the embryo becomes lengthened.

Before and also a short time after the entrance into the amnion, two important organs are connected with the embryo, which also lie external to the amnion, namely, the *vesicula erythroides* and the *vesicula umbilicalis.*

The vesicula erythroides or hitherto overlooked organ of the human ovum is a somewhat flat compressed bladder of a longish pear-shaped figure, the broader rounded end of which lies external upon the amnion over the lower part of the embryo; with its tapering extremities it passes toward the abdominal side of the embryo, and is bent forwards in a little angular projection. This in the eight to twelve days old ova is about three times as long as the embryo, and in the fourth week after conception is no longer visible. It is easily separated with the embryo from the external surface of the amnion, sometimes, however, it is more firmly fastened by its greater extremity to the surface of the amnion, and is difficult to separate.

The vesicula erythroides is transparent and of a milk-white colour; in its comparatively thick parietes, the naked eye can detect a great many red spherules scattered in all directions, which keep their red colour in spirits of wine. These little groups of spherules form into several broken lines; sometimes they form a double streak in which from the first, one can only clearly discover yellowish white spherules. Soon after the entrance of the embryo in the amnion cavity, each streak appears with a glass as if caused by the presence of a worm in the vesicula erythroides: under a powerful magnifying power, each of them appears to be subdivided.

By the gradual pressure of the embryo into the amnion cavity, the broader extremity of the vesicula erythroides separates itself from the amnion, follows the sinking in of the abdomen of the embryo into the vagina formed out of the amnion, fills up this vagina and thus is the vesicula erythroides in the human ovum on the umbilical cord. This happens in about the third week, and in naturally formed ova at this period the vesicula erythroides can be no longer found entire upon the amnion. But, instead of that, after the fourth and fifth week, the broad and obliterated end of the erythroides is seen as a white vesicle just opposite to the vesicula umbilicalis lying upon the amnion, and pressing on the vagina of the umbilical cord. From the vesicula erythroides two little canals pass into the embryo, and about the twentieth day all trace of the reddish streaks of the erythroides is lost, and from this time the abdomen of the fœtus, which was before flat, becomes rounded and protuberant.

The most attentive examination of the formation of the worm-like streaks, its entrance into the abdomen of the embryo, and its partial remaining in the umbilical cord as a real part of the cord, seems to me to leave no doubt that what Oken* before demonstrated in the other mammalia, also exists in man, namely, that the *intestines*, or at least a part of them, *are formed in the vesicula erythroides*, but in man these are not formed out of the sac of the erythroides, but in the proper cavity of the same.

The vesicula umbilicalis is the second organ connected with the embryo lying external to the amnion, and is also an essential part of the human embryo: it is a spherical bladder somewhat larger than the embryo, lies over the head of the embryo and is fastened loosely to the amnion. It is generally of a yellowish white colour, and sometimes perfectly transparent, containing a watery-like fluid, which does not become turbid in spirits or water; but I have never seen the least trace of the red spherules in it, nor could I ever discover any vessels within or on the outer side of the same. It gradually increases with the fœtus until the formation of the umbilical cord, and being then about two lines in diameter, it grows no more.

From the vesicula umbilicalis an exceedingly firm canal from one to three lines long, appearing to the naked eye like the finest silk, close by

* Oken, Isis, Veytrage, Heft j. Tab. iij. fig. 3. 10. 7.

the embryo, passes over into the vesicula erythroides, near which it becomes a little expanded. Immediately after the incipient formation of the intestines, one may see, with the aid of a strong magnifying glass, two very delicate threads arising from this canal, the one of which passes to the embryo, and the other in a contrary direction; but which, on account of its great fineness, I could not trace to its termination. Dr. P. also observed that as the amnion becomes distended, the vesicula umbilicalis leaves, or rather is drawn towards the umbilical cord, becomes gradually smaller, continues to a small white point by the side of the navel cord, and remains perceptible to the end of the third month. The fine canal abovementioned forms in the ninth week the vasa emphalomeseraica. Dr. Pockels thinks that both these organs, the vesicula umbilicalis and the vesicula erythroides are essential to the evolution of the fœtus: he attributes the death of the embryo in early abortion to the defective development or deficiency of these organs, and remarks, that out of thirty aborted, over which, according to this, some might be supposed to be two or three weeks old, only one in four ova were in all respects perfectly formed. Osiander has before made a remark to nearly the same effect in one of the commentations of the Royal Society of Gottingen: he says, that in the greater number of early miscarriages examined by him, he found the vesicula umbilicalis wanting.

Dr. Pockels' description of these delicate structures is exceedingly minute, and he has opened a field for much interesting anatomical and physiological enquiry.

---

41. *Case of Cæsarian Section in which both the Mother and Child were preserved.** Carolina Bechang was admitted into Graefe's Clinicum in the month of September, 1825, in the advanced stage of pregnancy. She was 30 years of age, four feet high, (Rhenish) and deformed by rickets.

The superior cristæ of the ossa ilii were distant from each other eleven inches; the umbilicus seven inches from the symphysis; the conjugate diameter was said by some to be only two inches and a quarter, whilst others made it two inches and a half. The diet of the patient was well regulated and rest strictly enjoined. Through fear of having an operation performed she left the Clinicum and returned home and could not be persuaded, through the most earnest entreaties, to return until she had been five days in labour; the pains were violent, the os uteri dilated, but the labour made no farther progress. On the 20th of September, she consented to be brought to the Clinicum and to have the operation performed, preparatory to which, she was bled to twelve ounces, had a clyster administered and the urine drawn off by the catheter.

This was performed by GRAEFE in the following manner.—A little after two o'clock, he placed his fore-finger of the left hand immediately

---

* De sectione Cæsarea in Instituto Clinico Chirurgico et Ophthalmiatrico Universitatis Literariæ Berolinensis hoc anno peracta, matre proleque superstitibus et salvis, Dip. May, 1825.

below the umbilicus, and, with a large scalpel, continued the incision from that point directly downwards in the linea alba to within one inch of the symphysis pubis, dividing, at this stroke, the entire parietes, the scalpel reaching also partially into the substance of the uterus. A second incision was immediately made in the same direction and of the same length into the body of the uterus, upon the placenta, which happened to lie on the fore part of its fundus, which, indeed, had been before predicted. The assistants now compressed firmly the edges of the divided abdominal parietes, upon the uterus, to prevent the protrusion of the intestines, which they succeeded in doing; and Graefe carried his hand in a moment, into the uterus, separated the placenta with his finger and thumb and then withdrew it and the child almost together. The child was very active and cried lustily. The uterus immediately and suddenly contracted, and the bleeding from it was inconsiderable, the quantity of blood lost did not altogether amount to more than twelve ounces. The operation was completed in four minutes and a half. The wound, which measured five inches and a half, was secured by three broad sutures and long straps of adhesive plaster, afterwards assisted by a bandage passed round the abdomen. No ligature was required in the operation. The child weighed six pounds and was well formed.

During the operation the patient nauseated and once slightly vomited; two hours after she complained of much pain in the wound; the abdomen felt hot, and was painful to the touch; the pulse full and frequent; she was again bled to twelve ounces. Ten drops of the aqua laurocerasi were given in a small draught, and this dose was repeated after the lapse of a few hours. At night, the pulse was again more frequent, about 110 in a minute, the dose of warm water was again given with a grain of the extract of hyoscyamus, which was afterwards altered. The patient passed a quiet night and slept a part of it.

On the following day a few drops of laudanum were directed to be given, and an injection of the infusion of chamomile flowers with linseed oil and salt to be administered. In the afternoon the patient felt very comfortable, the respiration was natural, the skin moist, and the pulse calmer. In the evening the symptoms became more unfavourable and the pain in the abdomen again severe and extended; bled to fifteen ounces. An infusion of chamomile flowers laid over the abdomen for a fomentation; a few spoonfuls of castor oil were given and an injection of a weak infusion of belladonna administered. The hyoscyamus and aqua laurocerasi were alternately taken, the lochia continued to be discharged. The patient again passed a quiet night, was quite composed, the temperature of the skin moderate, and the pulse soft. Things went on thus favourably till the fifth day after the operation, when the dressings were partially removed; the bowels were emptied by common clysters. On the third day it was seen by a removal of the upper part of the dressings that the edges of the wound were in good apposition. On the fourth, the upper ligature was removed; and, on the fifth, that part of the wound lying above the third suture was united. On the ninth day the entire wound, excepting towards the symphysis, had firmly united and the two lower ligatures were taken away. The patient continued to take, from time to time, sedative medicine, and was allowed only a strictly spare diet until this time, when she was permitted to take a small quantity of soup and a little red wine. The lochia continued to be discharged regularly, and, in three weeks, the patient was so well, that she was allowed to sit up a little; this she did daily, and, in another three weeks was perfectly sound. In the early part of the month of November the patient returned with her child in perfect health to her former occupations.

---

42. *Case of a complete Prolapsus of the Uterus during Pregnancy and Labour.* A woman, about 38 years of age, was, after delivery of her third child, for almost a year, troubled with a partial prolapsus of the vagina, which was at last removed by using a pessary. Soon after she became again pregnant, and, in the third month, in consequence of having made some sudden exertion, the prolapsus vaginæ returned as before. In this state she continued even to the end of her pregnancy without having adopted any measures for her relief; the prolapsus gradually increased in size, so that two months before the time of delivery it was as large as a full-grown child's head; painful, and always accompanied with the sensation of a dragging weight from the loins. When the patient wished to sit down she was obliged to sit between two stools. About three weeks before her expected time of confinement she fell down, the protrusion suddenly increased in size, some of the liquor amnii escaped, and when the midwife arrived she found a foot presenting through the tumour. A lithographic sketch accompanies this case from which it appears that the tumour reached half way down the thigh, and in circumference, was about the size of the thigh. The child was discovered to be dead; the orifice of the uterus was sufficiently dilated in a short time to allow of the delivery of all the child, with the exception of the head, which was obliged to be perforated. The prolapsed parts were afterwards carefully returned, and by the diligent use of astringents persevered in for two months the evil was permanently removed.—*Siebold's Journal für Geburtshulfe, &c.* 1826.

---

43. *Extirpation of an Uterus completely inverted, and affected with Fungus Hæmatodes, successfully performed by Dr. Rheineck of Memmingen.*—A married woman, 43 years of age, who had never borne any children was, in the month of December, 1824, attacked with fever, from which she slowly recovered. The weakness of the patient was very great, and a prolapsus of the uterus, which gradually became inverted, followed, attended with frequent hæmorrhage and discharge, so that she was almost worn to the grave. During the progress of the disease she had felt great pain in the belly and sudden and violent spasms. Dr. Rheineck found the whole of the uterus inverted and without the labia externa; its surface was loose, fungous, and easily broken down upon pressure in several places; but there was no hardening nor ulceration in any part of it. The irritation which this prolapsed tumour occasioned was very great, so that the life of the patient was endangered. A careful examination was instituted to discover whether this structure was merely an excrescence or really the degenerated uterus; by which it was rendered quite clear that the whole of the uterus was inverted. It was agreed in consultation to apply a broad ligature round its base, for which purpose the lower part of the tumour was laid hold of, drawn a little forward, and a firm ligature, secured with a double surgeon's knot, applied. In about three weeks the part had separated, and the part above which the ligature was tied, completely cicatrized; during this interval the patient was several times very dangerously ill, and was only rescued by great care and attention.

The operator had before performed a similar operation, in which case the patient died suddenly from hæmorrhage at the separation of the ligature. Osiander, Struve, Langenback, Sauter, Siebold and Zoug, have, in late years, performed the same operation with various degrees of success.—*Siebold's Journal für Geburtshulfe, &c.* 1826.

